# Vegan and Omnivorous High Protein Diets Support Comparable Daily Myofibrillar Protein Synthesis Rates and Skeletal Muscle Hypertrophy in Young Adults

**DOI:** 10.1016/j.tjnut.2023.02.023

**Published:** 2023-02-22

**Authors:** Alistair J. Monteyne, Mariana O.C. Coelho, Andrew J. Murton, Doaa R. Abdelrahman, Jamie R. Blackwell, Christopher P. Koscien, Karen M. Knapp, Jonathan Fulford, Tim J.A. Finnigan, Marlou L. Dirks, Francis B. Stephens, Benjamin T. Wall

**Affiliations:** 1 Department of Public Health and Sports Sciences, Nutritional Physiology Research Group, University of Exeter, Exeter, United Kingdom; 2 Department of Surgery, University of Texas Medical Branch, Galveston, Texas, United States; 3 Sealy Center of Aging, University of Texas Medical Branch, Galveston, Texas, United States; 4 College of Medicine and Health, University of Exeter, Exeter, United Kingdom; 5 Marlow Foods Ltd, Stokesley, North Yorkshire, United Kingdom

**Keywords:** mycoprotein, muscle protein synthesis, hypertrophy, resistance exercise, vegan

## Abstract

**Background:**

It remains unclear whether non–animal-derived dietary protein sources (and therefore vegan diets) can support resistance training-induced skeletal muscle remodeling to the same extent as animal-derived protein sources.

**Methods:**

In Phase 1, 16 healthy young adults (*m = 8*, *f = 8*; age: 23 ± 1 y; BMI: 23 ± 1 kg/m^2^) completed a 3-d dietary intervention (high protein, 1.8 g·kg bm^−1^·d^−1^) where protein was derived from omnivorous (OMNI1; *n* = 8) or exclusively non-animal (VEG1; *n* = 8) sources, alongside daily unilateral leg resistance exercise. Resting and exercised daily myofibrillar protein synthesis (MyoPS) rates were assessed using deuterium oxide. In Phase 2, 22 healthy young adults (*m = 11*, *f = 11*; age: 24 ± 1 y; BMI: 23 ± 0 kg/m^2^) completed a 10 wk, high-volume (5 d/wk), progressive resistance exercise program while consuming an omnivorous (OMNI2; *n* = 12) or non–animal-derived (VEG2; *n* = 10) high-protein diet (∼2 g·kg bm^−1^·d^−1^). Muscle fiber cross-sectional area (CSA), whole-body lean mass (via DXA), thigh muscle volume (via MRI), muscle strength, and muscle function were determined pre, after 2 and 5 wk, and postintervention.

**Objectives:**

To investigate whether a high-protein, mycoprotein-rich, non-animal-derived diet can support resistance training-induced skeletal muscle remodeling to the same extent as an isonitrogenous omnivorous diet.

**Results:**

Daily MyoPS rates were ∼12% higher in the exercised than in the rested leg (2.46 ± 0.27%·d^−1^ compared with 2.20 ± 0.33%·d^−1^ and 2.62 ± 0.56%·d^−^^1^ compared with 2.36 ± 0.53%·d^−1^ in OMNI1 and VEG1, respectively; *P <* 0.001) and not different between groups (*P >* 0.05). Resistance training increased lean mass in both groups by a similar magnitude (OMNI2 2.6 ± 1.1 kg, VEG2 3.1 ± 2.5 kg; *P >* 0.05). Likewise, training comparably increased thigh muscle volume (OMNI2 8.3 ± 3.6%, VEG2 8.3 ± 4.1%; *P >* 0.05), and muscle fiber CSA (OMNI2 33 ± 24%, VEG2 32 ± 48%; *P >* 0.05). Both groups increased strength (1 repetition maximum) of multiple muscle groups, to comparable degrees.

**Conclusions:**

Omnivorous and vegan diets can support comparable rested and exercised daily MyoPS rates in healthy young adults consuming a high-protein diet. This translates to similar skeletal muscle adaptive responses during prolonged high-volume resistance training, irrespective of dietary protein provenance.

This trial was registered at clinicaltrials.gov as NCT03572127.

## Introduction

Resistance exercise [[1], [2], [3]] and dietary protein ingestion [[4], [5], [6], [7]] both increase mixed (MPS) and myofibrillar (MyoPS) protein synthesis rates. The additive effect of these two anabolic stimuli repeated over time allows for substantive muscle protein accrual and, therefore, an increase in muscle fiber size [8]. This is the physiological and nutritional basis for resistance training-induced muscle hypertrophy and, at least in part, muscle strength.

It has been established that training-induced gains in muscle mass and strength can be augmented by adhering to a high-protein diet, well above the United Kingdom/United States RDA of 0.75–0.8 g·kg bm^−1^·d^−1^ [9], with 1.6 g·kg bm^−1^·d^−1^ approaching optimal [10]. The basis of such recommendations is largely obtained from studies where participants undergoing resistance training were supplemented with animal-derived dietary protein sources (for example, whey, casein, or milk) and MPS/MyoPS rates and/or muscle mass and strength were determined alongside an omnivorous diet [4,11,12]. It is therefore unclear whether current dietary protein recommendations to support optimal resistance training adaptation can be appropriately applied to those adhering to vegan diets. This is pertinent given the increasing drive, both at a societal and governmental level, to reduce the consumption of animal products [13].

It has been suggested that plant-based dietary protein sources are typically inferior in their capacity to support resistance training adaptations [14]. In support, soy protein isolate ingestion (22.2-g) stimulates a lower post-exercise MPS response than an isonitrogenous bolus of whey protein (21.4-g) [15] and, accordingly, supports inferior hypertrophy when supplemented during prolonged training in comparison to dairy protein [16,17]. In contrast, comparable postprandial MyoPS rates were observed after the ingestion of ample doses (30-g) of wheat or potato protein isolate and milk protein [18,19]. Although minimal MPS/MyoPS data are available for other non–animal-derived protein sources, pea protein isolate supplementation has been reported to facilitate comparable resistance training adaptation to that of whey protein supplementation in subjects consuming a high protein omnivorous diet [20]. Moreover, a recent resistance training intervention study by Hevia-Larraín et al. [21] demonstrated that habitual vegans increased muscle mass and strength to a similar degree as a habitually omnivorous group during lower body training.

We recently demonstrated that mycoprotein (a non-animal, fungal-derived dietary protein source) robustly stimulates post-exercise MPS rates [22], and is feasibly incorporated into high-protein vegan diets (as supplements and food products) to support comparable daily post-exercise MyoPS rates as omnivorous diets in older adults [23], making it a suitable protein choice for longer-term vegan diets designed to optimize training adaptations. In the present study, we hypothesized that young adults consuming a high-protein, mycoprotein-rich, and virtually exclusively vegan intervention diet would exhibit comparable daily (post-exercise) free-living MyoPS rates and comparable increases in muscle fiber size, muscle volume, whole-body lean mass, and strength compared with an isonitrogenous omnivorous diet during high-volume, high-intensity, whole-body progressive resistance exercise training.

## Methods

### Participants

Participants were recreationally active and had resistance exercise experience, having formally completed structured resistance exercise training regimens for extended periods of time (structured training of 6 mo and within the earlier 3 y), although a degree of heterogeneity in participant training experience was inevitable. Participants were habitually omnivorous, with the exception of a single vegan participant in Phase 1 of the study, and 2 vegan participants in Phase 2 of the study. All participants were informed of the nature and possible risks of the experimental procedures before providing written informed consent. Participants attended the laboratory for a medical screening, wherein height, body mass, and blood pressure were measured, as well as a general medical questionnaire completed, to assess their eligibility for participation and to confirm the absence of adverse health conditions. Participants were deemed healthy based on their blood pressure (≤140/90 mmHg), BMI (18–30 kg/m^2^), and the absence of any diagnosed metabolic conditions, cardiovascular disease, or motor disorders. The eligibility criteria did not differ between the respective phases of the study. Participants also completed the International Physical Activity Questionnaire (IPAQ) [24]. Participants recorded habitual nutritional intake for 3 d (2 weekdays and a weekend day) either before or shortly after completing the study. This study was conducted according to the guidelines laid out in the Declaration of Helsinki, and all procedures involving human subjects/patients were approved by the NHS Health Research Authority Research Ethics Committee (18/LO/0374) and registered as a clinical trial with clinicaltrials.gov (NCT03572127). Participants were recruited between May 2018 and October 2019, and data were collected between May 2018 and March 2020.

### Experimental design

**FIGURE 1:**
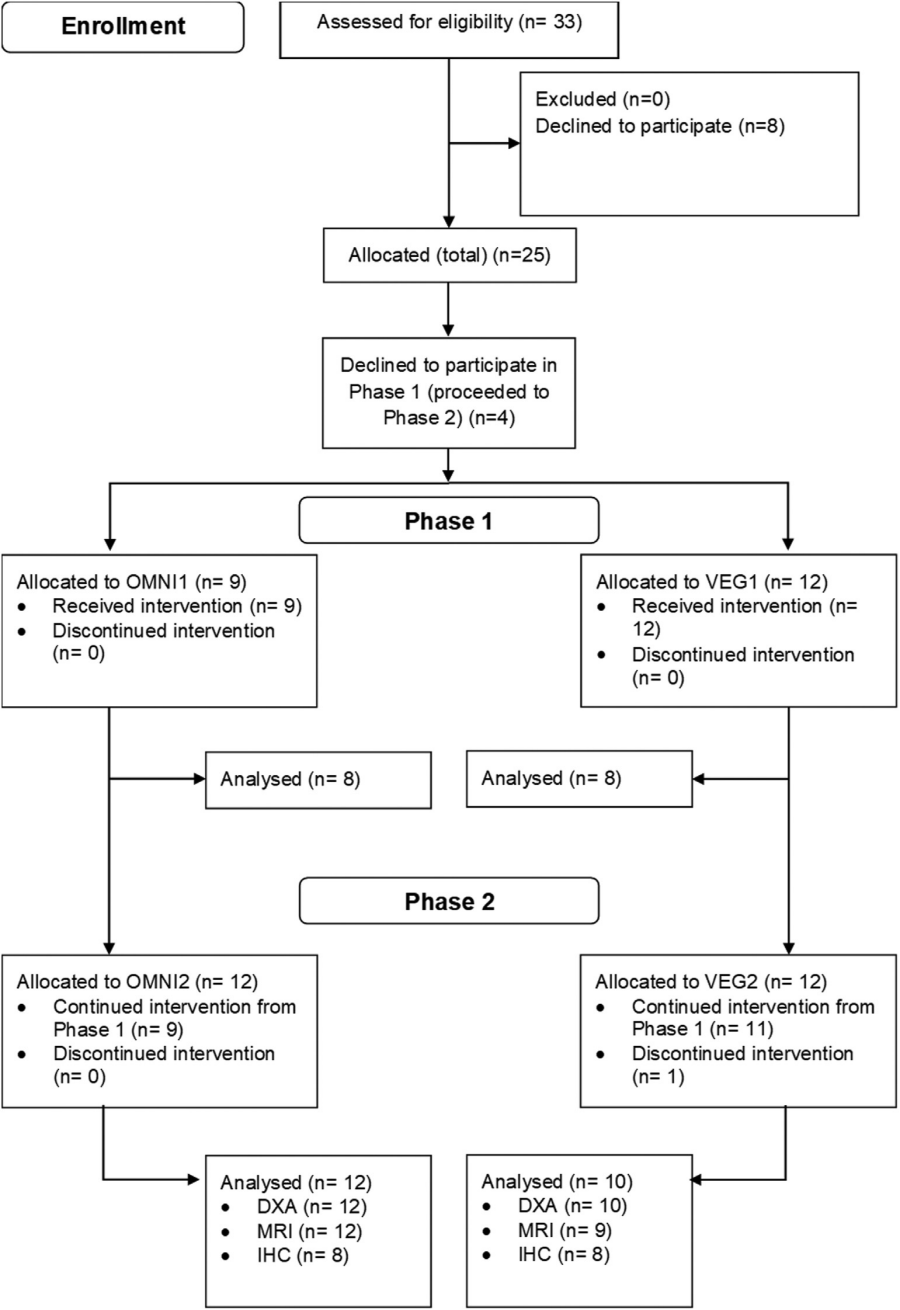
Flow chart of participant recruitment and allocation within the 2 phases of the experimental protocol. IHC, immunohistochemistry; OMNI, omnivorous; VEG, vegan.

**FIGURE 2:**
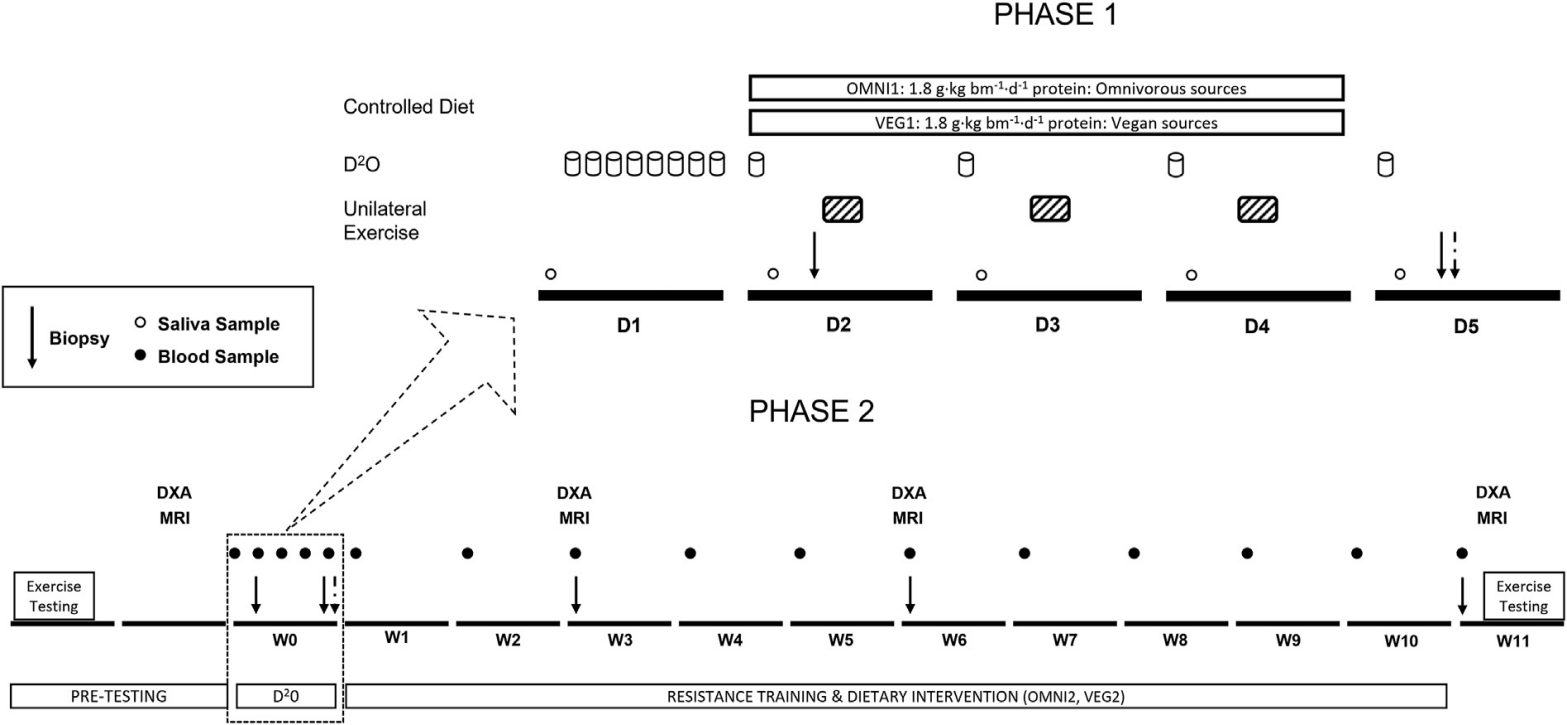
Schematic representation of the study protocol. Phase 1, 16 healthy young adults consumed a 3-d fully controlled, eucaloric, and high-protein (1.8 g·kg bm^−1^·d^−1^) diet, where the protein was provided predominantly from animal (OMNI1; *n* = 8) or exclusively non-animal (VEG1; *n* = 8) sources. During the dietary control period (days: 2–4) participants conducted a single bout of unilateral isokinetic knee extension exercise (5 × 30 contractions) each morning. On day 1, participants consumed 400 mL deuterated water with 50 mL doses consumed daily thereafter. Saliva samples were collected daily, and muscle biopsies were collected from both the rested (straight arrow) and exercised (dashed arrow) legs to determine daily myofibrillar protein synthesis rates. Phase 2, 22 healthy young adults completed a 10-wk high-volume resistance exercise training program, while consuming a high-protein omnivorous diet (OMNI2; *n* = 12) or a majority non–animal-derived diet (VEG2; *n* = 10). Participants underwent DXA and MRI scans, muscle biopsies, and strength testing, at regular intervals to characterize resistance exercise-induced muscle adaptations. “D1,” “D2,” etc. refer to day 1, day 2, etc.; “W0,” “W1,” etc. refer to week 0, week 1, etc. OMNI, omnivorous; VEG, vegan.

Experimental testing was divided into 2 phases, with participants assigned to 1 of the 2 parallel dietary intervention groups by the lead investigator, using simple randomization, which differed only with respect to the primary sources of dietary protein consumed throughout the study: omnivorous (OMNI) or vegan (VEG). Participants who continued involvement from Phases 1 to 2 of the study retained their initial randomization from Phase 1. Four participants declined to participate in Phase 1 of the study, but were assigned to Phase 2 of the study in an attempt to increase the sample size to account for attrition, potential insufficient muscle sample size, and/or loss of samples during analysis. The study was an open-label design, therefore, it was not practically possible to blind the participants or the investigators. Analysis was performed in a blinded manner, with the exception of body composition and strength data in which the real-time nature of these measures precluded blinding. If a participant’s habitual dietary choices (that is, vegan) precluded their inclusion in the omnivorous group, they were assigned to the vegan group (and therefore not randomized) (*n* = 1 and 2 in Phases 1 and 2, respectively). A graphical representation of participant allocation, passage through the study, and experimental study design is provided in FIGURE 1, FIGURE 2, respectively.

To allow for a controlled assessment of dietary protein source on free-living, daily MyoPS rates in line with our previous work [23,25], Phase 1 of the study consisted of 3 d of full dietary control with the application of deuterium oxide tracers. The OMNI1 group was provided with predominantly animal-derived protein sources including milk protein supplementation, and the VEG1 group with exclusively non–animal-derived protein sources including mycoprotein-containing meals and supplements.

Phase 2 examined how the dietary protein source affects the longer-term muscle adaptive responses and consisted of 10 wk of dietary manipulation alongside 5 × per wk-structured resistance exercise. The participants in the OMNI2 group were counseled to consume a high-protein omnivorous diet, including milk protein supplementation, and those of the VEG2 group consumed an exclusively non–animal-derived diet, including mycoprotein supplementation. Muscle fiber cross-sectional area (CSA), DXA whole-body lean mass, and MRI thigh muscle volume were determined pre, after 2 and 5 wk, and post-intervention, with muscle strength and muscle function determined pre and post-intervention.

### Pretesting

After screening and admittance to the study, all participants underwent a period of pre-testing, which took place ≥5 and ≤10 d before the initiation of the experimental period. This included strength testing, muscle function testing, and familiarization with the exercise equipment to be used. Participants completed 1 repetition maximum (1RM) tests for the barbell back squat, conventional deadlift, and incline (30°) barbell bench press. Participants then completed a number of unilateral leg exercise tests on an isokinetic dynamometer (Biodex Medical Systems, Shirley, New York, USA), in line with our previous work (See Supplemental Material for further detail) [22].

#### Phase 1

##### Experimental protocol

Participants were asked to refrain from alcohol and caffeine consumption, as well as from strenuous exercise for 2 d before and throughout the experimental protocol, though to keep all other daily habitual activities as normal. Participants attended the laboratory each day for a 5-d (Monday to Friday) experimental period with days 2 to 4 (that is, 3 d, Tuesday to Thursday, inclusive) involving dietary control. During each day of the 3-d dietary control period, participants attended the laboratory to conduct a single bout of unilateral leg extension exercise consisting of 5 sets of 30 repetitions of maximal concentric isokinetic knee extension contractions of their dominant leg (see *pretesting* section for details) and were then provided with their food for the day. To measure daily MyoPS rates, participants underwent a deuterium oxide dosing protocol (described below) in line with our previous work [25,26], and muscle biopsies were collected before commencing the controlled diet [that is, Tuesday ∼0800 h; single muscle biopsy from the (to-be) rested leg] and immediately after (that is, Friday ∼0800 h; bilateral biopsies from the rested and exercised legs). Muscle biopsies were obtained under local anesthesia, using a percutaneous Bergstrom biopsy needle technique [6], from the *m. vastus lateralis* approximately 15 cm above the patella and approximately 3 cm below the fascia. Muscle tissue was quickly assessed, and any blood or non-muscle tissue was dissected and discarded. The majority of each biopsy sample was immediately frozen in liquid nitrogen, with a single aliquot set on a glass slide, placed on embedding medium, and frozen in liquid isopentane at −160 °C. All muscle samples were then stored at −80 °C until further analysis.

##### Dietary intervention

Basal metabolic rate (BMR) was estimated using the Henry equations based on age, gender, and body mass [27]. Individual energy requirements were then calculated by multiplying the participant’s BMR by their IPAQ-derived PAL. Thereafter, an individual 3-d meal plan was designed for each participant with all food prepared, weighed, and packaged in-house in the Nutritional Physiology Unit’s research kitchen facility. Nutritional information for the 2 diets is provided in Table 2.

**TABLE 1:** Baseline participants’ characteristics

	Phase 1			Phase 2	
	OMNI1	VEG1	*P*	OMNI2	VEG2
	(n = 8)	(n = 8)		(n = 12)	(n = 10)
	3 f	2 f		6 f	5 f
	5 m	6 m		6 m	5 m
Age (y)	24 ± 7	22 ± 5	0.890	24 ± 6	24 ± 6
Body mass (kg)	73 ± 10	70 ± 9	0.537	71 ± 11	69 ± 10
Height (cm)	175 ± 13	175 ± 10	0.970	173 ± 11	172 ± 10
BMI (kg·m^−2^)	24 ± 3	23 ± 2	0.403	24 ± 3	23 ± 2
Fat (% body mass)	25 ± 11	23 ± 9	0.538	25 ± 9	23 ± 9
Lean mass (kg)	51 ± 14	49 ± 11	0.923	51 ± 12	51 ± 12

Values represent mean ± SD. No differences between groups (all *P* > 0.05). Independent sample t-tests were used to compare characteristics across groups. f, female; m, male; OMNI, omnivorous; VEG, vegan.

**TABLE 2:** The nutritional content of the participants’ habitual diets and of the intervention diets during Phase 1 of the study

A	OMNI1	VEG1	*P*	
	(*n* = 8) 3 f 5 m	(*n* = 8) 2 f 6 m	Group effect	Intervention effect
Habitual diet				
Energy (MJ·d^−1^ (kcal·d^−1^))	10.7 ± 2.2 (2,559 ± 526)	10.8 ± 3.6 (2,584 ± 850)	0.950	—
Protein (g·d^−1^)	121 ± 43	108 ± 38	0.568	—
Protein (g·kg bm^−1^·d^−1^)	1.6 ± 0.4	1.6 ± 0.5	0.985	—
CHOs (g·d^−1^)	267 ± 51	327 ± 110	0.223	—
Fat (g·d^−1^)	102 ± 30	90 ± 37	0.557	—
Fiber (g·d^−1^)	28 ± 15	41 ± 15	0.138	—
Intervention diet				
Energy (MJ·d^−1^ (kcal·d^−1^))	11.3 ± 1.7 (2,707 ± 394)	11.5 ± 1.8 (2,755 ± 433)	0.818	0.505
Protein (g·d^−1^)	131 ± 20	127 ± 16	0.671	0.233
Protein (g·kg bm^−1^·d^−1^)	1.8 ± 0.0	1.8 ± 0.0	N/A	0.084
CHOs (g·d^−1^)	356 ± 52	363 ± 71	0.813	0.024
Fat (g·d^−1^)	76 ± 12	77 ± 11	0.885	0.024
Fiber (g·d^−1^)	36 ± 5	73 ± 8	<0.0001	0.001

Values represent mean ± SD. Participants received a 3-d controlled, eucaloric, and high-protein diet, alongside daily unilateral resistance exercise, with deuterated water used to measure rested and post-exercise daily myofibrillar protein synthesis rates. In OMNI1, participants consumed an omnivorous diet with the majority of dietary protein coming from animal-derived sources. In VEG1, participants consumed a diet derived from non-animal sources with a large proportion of their protein coming from mycoprotein-containing products and mycoprotein. Both groups received 1.8 g·kg bm^−1^·d^−1^ protein. Dietary data were analyzed with repeated measures 2-factor ANOVA. f, female; m, male; OMNI, omnivorous; VEG, vegan.

Subjects consumed a diet containing 1.8 g of protein per kg of body mass (bm) per day (g·kg bm^−1^·d^−1^) with 24%–27% of energy being provided by fat and 50%–55% from carbohydrates in OMNI1, and 22%–27% and 48%–58% of energy being provided by fat and carbohydrates, respectively, in VEG1 (variation due to different energy requirements and the matching of protein intake). The meals [3 meals per day (breakfast, lunch, and dinner), alongside snacks] were identical between the 2 groups, aside from meat or dairy providing the primary protein source in lunches and dinners for the OMNI1 group and this being replaced by mycoprotein containing products (Quorn Foods) in the VEG1 group. The OMNI1 group received 39-g supplemental milk protein daily (31-g protein, 2-g carbohydrate, <1-g fat, 131 kcal), and the VEG1 group 70-g supplemental mycoprotein (31-g protein, 7-g carbohydrate, 9-g fat, 232 kcal) to drink before sleep, with this dosing strategy applied to match protein content within the supplements between groups. A small amount of mycoprotein was also added to the breakfast in the VEG1 group to more closely equate the protein in the breakfast meal between conditions. Breakfast was consumed within 1 h of completing the unilateral resistance-type exercise and provided 18 ± 3 and 20 ± 5-g protein per day in OMNI1 and VEG1, respectively. The participants in OMNI1 group consumed meals based on chicken, pork, and dairy. In the VEG1 group, this was substituted for mycoprotein-containing vegan product substitutes. A document and diary detailing the plan were provided to the subjects to log mealtimes and provide recipe information/instructions. Participants’ body mass was measured wearing light clothing at the start and end of the 3-d controlled diet period (seca 703 column scale, seca GmbH & Co. KG, Hamburg, Germany). Each morning, the researchers discussed with the participants any questions or issues that may have arisen, before the next day of food was provided.

##### Deuterated water dosing protocol

The deuterated water (D_2_O) dosing protocol was based on our previous work [23,26] and that of others [28]. Day 1 (Mon) of the experimental protocol acted as a D_2_O loading day where participants consumed 400 mL of 70% D_2_O separated over the day as 8 × 50 mL boluses (CK Isotopes Ltd). Upon arrival at the laboratory (0730 h) background saliva samples were collected before the first bolus of D_2_O was ingested. The first dose of D_2_O was consumed at ∼0800 h with the remaining loading doses being consumed every 1 h (doses 2 and 3) and then every 1.5 h thereafter. Participants stayed at the laboratory until 4 out of the 8 loading day doses had been consumed, with the remaining doses being consumed at home under the instruction of timings. Every day after the loading day, participants consumed a maintenance dose of D_2_O (50 mL) upon waking (∼0800 h). At least 90 min (∼09:30 h) after the daily D_2_O maintenance dose, a saliva sample was collected using a cotton mouth swab (Celluron), for the calculation of average enrichment over the measurement period to be used as a representative precursor pool.

##### Biochemical analyses

Body water deuterium enrichments (from saliva samples), and myofibrillar bound ^2^H alanine enrichments, extracted from ∼50 mg of wet weight muscle tissue, were analyzed at the University of Texas Medical Branch using IRMS and GC-MS, respectively, as previously reported (details in Supplemental Information) [23].

##### Calculations

Daily MyoPS rates were calculated [expressed as fractional synthesis rates (FSR)] based on the incorporation of the mean body water deuterium enrichment over the 3-d intervention as a precursor pool into myofibrillar bound proteins [26,28,29]. FSR was calculated using the standard precursor-product method and expressed as daily rates as follows:

$$FSR\;\left(\%\;\cdot {\text{day}}^{-1}\right)=\left(\frac{E_{m2}-E_{m1}}{E_{precursor}\;x\;t}\right)x\;100$$

[where *E*_m1_ and *E*_m2_ are the myofibrillar muscle protein-bound enrichments pre (one leg only) and post (either the rested or exercised leg) the dietary intervention. *E*_precursor_ represents mean body water deuterium enrichment corrected by a factor of 3.7. *t* represents the time between biopsies (that is, 3 d)].

#### Phase 2

##### Resistance exercise training program

Participants completed 5 exercise sessions per wk in a program designed to maximize muscle hypertrophy over a 10-wk period. The training was structured in a “pull-push-lower” pattern, training each major muscle group twice per wk (Supplemental Table 1). The 2 weekly rest days were timed at the participants own discretion/convenience, although it was advised that they rest on the fourth and seventh day of each wk, where possible, to optimize recovery. The first 5 exercise sessions were fully supervised in the laboratory gymnasium, allowing proper technique to be taught and discussed. Thereafter, participants were permitted to complete exercise sessions at the laboratory gym or their regular gymnasium. Participants were required to visit the laboratory facility, or were visited in their chosen facility, at a minimum of every fortnight (more so when required) so that a researcher was able to monitor their progress and technique, and discussion was also conducted on training diaries on a bi-weekly basis. Participants completed a training diary where they were asked to record load, repetitions, and any other pertinent information. Participants trained 5 times per wk, aiming for a total of 50 sessions, for a period of 10 ± 1 wk. If participants missed training sessions in a wk, due to illness, injury, or unforeseen circumstances, they were required to catch up on those sessions up at a later point. If participants were to miss >50% of sessions over a 2-wk period, they were excluded from the study. Participants completed an average of 47 ± 4 (range: 37–50) and 47 ± 4 (range: 37–50) sessions (*P* = 0.91 between groups) over a period of 10 ± 1 (range: 8–11) and 10 ± 1 (range: 9–11) wk (*P* = 0.37 between groups) in the OMNI2 and VEG2 groups, respectively.

##### Dietary intervention

While undertaking the resistance exercise training program, participants were advised upon a dietary intervention to support muscle hypertrophy with respect to sufficient daily energy intake [[30], [31], [32], [33], [34]] and dietary protein amount [10], quality [35], and timing [36,37]. Based upon current recommendations, the omnivorous control diet acted as a model; of what we might consider a (close to) optimal nutritional approach. We then matched the VEG2 group to this model, with the exception of protein source, to test the hypothesis that this would impair an “optimal” approach to training and nutrition to support the muscle adaptive response.

Participants were provided with a caloric target [(BMR × PAL) × 1.1] to place them in a ∼0 to 10% energy surplus, and a protein target of 2 g·kg bm^−1^·d^−1^, this aimed to account for modest underachievement of targets and for daily dietary fluctuations, without regularly falling below “optimal” [[30], [31], [32], [33], [34],^38^]. Participants in OMNI2 were instructed to consume an omnivorous diet, focusing their intake on high-quality animal-derived proteins (that is, meat, milk, yogurt, cheese) [35]. Participants in VEG2 were instructed to avoid animal products for ≥6 d per wk [although most (*n =* 7 of 10) adopted an exclusively vegan diet, and (*n =* 1 of 10) only consumed dairy in coffee], focusing their intake on protein-rich non–animal-derived foods (for example, mycoprotein-containing products, pulses, soy). To facilitate reaching protein intake targets, the research team provided participants with a weekly supply (∼1–2 products per day) of chicken or beef (OMNI2) or high mycoprotein-containing vegan products (VEG2) (∼1–2 products per day) to be used as the main protein source for some meals. The OMNI2 group also received 59-g supplemental milk protein daily (47-g protein, 2-g carbohydrate, <1-g fat, 198 kcal, 19.5-g to drink post-training to maximize post-exercise MyoPS rates [4,5], and 39-g to drink before sleep to promote higher overnight MyoPS rates, recovery, and optimize protein distribution) [36,37,39]. The VEG2 group instead received 105-g mycoprotein (46-g protein, 10-g carbohydrate, 13-g fat, 348 kcal), 35-g post-training, and 70-g before bed. On non-training days, the post-training dose was consumed between meals. Finally, to provide a (close to) optimal nutritional approach to supporting muscle hypertrophy, and to minimize the risk of dietary deficiency in the VEG2 group [40,41] (thus obscuring any effects of dietary protein source *per se*), all participants also supplemented with creatine monohydrate (Myprotein) throughout the study, consuming 5 × 5-g doses for 5 d during wk 1 (that is, loading), and 5-g per day as a maintenance dose thereafter [42,43].

Participants were required to record their dietary intake for a minimum of 3 d per wk, but were requested to record as many days as possible. All dietary recordings were performed via MyFitnessPal to allow for ease of recording and real-time feedback on energy and macronutrient intake compared with targets. Dietary data were discussed weekly with the research team, and further advice/encouragement was offered if daily targets were not being met (with participants excluded if they fell below 1.6 g·kg bm^−1^·d^−1^ dietary protein for consecutive wks).

##### MRI

A 1.5 tesla (T) MRI scanner (Intera, Phillips) was used to obtain images of the right thigh in the axial plane over the full length of the femur. A T1-weighted 3D turbo spin echo sequence was used (field of view, 500 × 500 mm; reconstructed matrix, 512 × 512 mm; echo time, 15 ms; repetition time, 645 ms; slice thickness, 5 mm; slice gap, 5 mm) with the subject lying still in the supine position. A 4-element sense body radiofrequency coil was wrapped around both the thighs. During the pretesting scan, a specified distance from a bony landmark (the lateral and medial femoral condyles) in the frontal plane was used to center the axial plane images [44]. This same distance was used on all subsequent MRI scans to ensure that the axial images were in the same location along the length of the thigh on all scans. 3D Slicer MRI software (Slicer 4.10.2; www.slicer.org/software) was used to analyze the images obtained in the axial plane. On average ∼45 images were acquired along the length of the femur, with the bottom 25% (from the lateral femoral condyle working proximally) and top 25% (from the greater trochanter working distally) excluded, in line with previous work from our laboratory [25,45]. The remaining images, in the central 50% portion of the thigh, were processed using a combination of automated thresholding, manual thresholding, and manual segmentation as reported previously [25]. Briefly, individual slices of the whole thigh were thresholded automatically to provide an approximate outline of the thigh musculature, before being further processed to produce a precise final image. The same experimenter performed all manual segmentation of the images to reduce variability in processing, and therefore ensure consistency across the data set. Thigh muscle volume was automatically calculated using 3D Slicer’s segment statistics function. Subsequently, quadriceps, hamstrings, and adductor muscles were delineated and manually segmented, before the volume was calculated in the same manner as above.

##### DXA

Participants underwent a whole-body DXA scan (E Lunar Prodigy Healthcare Corp, Madison, WI, USA, 2006) before testing and after 2 wk, 5 wk, and upon completion of the intervention, to establish changes in body composition. Participants were scanned at ∼08:00 following a 10–12 h overnight fast with no fluid intake that morning in line with current recommendations to minimize the contribution of altered fluid balance [46], and their bladder voided. Body position was recorded with the use of bony landmarks and scan table references to ensure that body position was maintained as constant as possible between scans. All participants were scanned, in standard mode, with their hands by their sides in a supinated position with their feet held ∼10 cm apart.

##### Immunohistochemistry

Optimal cutting temperature compound (OCT) embedded muscle samples were transversely orientated on a cutting mount and sectioned at −20 °C (8 μm thickness) on a Cryostat NX70 cryostat (Thermo Scientific, Mass. US), and then mounted on glass slides. Samples were air-dried for 30 min, fixed with 4% paraformaldehyde (15 min), washed (3 × PBS), blocked (60 min), washed (3 × PBS), and incubated (2 h) at room temperature with primary antibodies against laminin (2E8 MIgG2a kappa light chain, 1:100 in PBS, Developmental Studies Hybridoma Bank) and myosin heavy chain type 1 (slow isoform) (A4.84 MIgM0, 1:100, Developmental Studies Hybridoma Bank). After washing (3 × PBS), samples were incubated (1 h) in the dark at room temperature with appropriate secondary antibodies: MHC secondary (Goat anti-mouse IgM mu-chain, 1:200, Alexa 647, abcam), and laminin secondary (Goat anti-mouse IgG2a gamma-chain, 1:250, Alexa 568, Thermo Fisher Scientific). Samples were then washed (3 × PBS), dried, and mounted with cover glasses using Mowiol. All images were captured digitally (LAS X software; Leica Microsystems GmbH) using a Leica DMi8 S widefield fluorescence microscope (Leica Microsystems GmbH) coupled to a Hamamatsu C11440-22C camera (Hamamatsu Photonics, Shizuoka, Japan) at ×20 magnification [Epifluorescence signal was recorded by using excitation filters for laminin (Texas Red, 540–580 nm), and MHC-I (Y5, 620–650 nm)]. Fiber type and mean fiber CSA were obtained using the MuscleJ macro [47] on the Fiji software platform [48]. After quantification, automatically selected fibers were manually inspected and verified, with any misidentified fibers removed from the subsequent analysis. On average, 3.6 ± 0.7 cryosections or images were analyzed per participant [Pre (*n =* 16); W2 (*n =* 13); W5 (*n =* 14); Post (*n =* 15)].

##### Statistics

A 2-sided power analysis based on previous research [28] showed that 8 per group was sufficient to detect the expected differences in MyoPS rates between rested and exercised legs when using a 2-factor ANOVA (*P* < 0.05, 95 % power, *f* = 1·68; G∗power version 3.1.9.2). A secondary 2-sided power analysis, considering previous research [16,49], showed that 11 per group was sufficient to detect expected differences in muscle mass between interventions when using a 2-factor ANOVA (*P* < 0.05, 80 % power, *f* = 0.25; G∗power version 3.1.9.2). All data are presented as mean ± SD, and all statistical analyses were conducted in GraphPad Prism version 7.0 (GraphPad Software). Missing data analysis (regression imputation) was used minimally where necessary, and in cases where there were significant missing data within a participant for a given variable, they were excluded from that analysis. Data were tested for sphericity, and where violations occurred, the Greenhouse-Geisser correction was automatically applied. Violations of normality were tested for using the Shapiro–Wilk test. Independent samples *t*-tests were used to compare each of the participants’ characteristics across groups, and dietary data where appropriate. Dietary data were also subjected to repeated measures 2-factor ANOVAs, in both phases, to compare the differences between habitual and intervention diets.

For Phase 1, a repeated measures 2-factor ANOVA was used to compare body mass [group (OMNI1 compared with VEG1) and time (pre compared with post)] and a mixed-effects model ANOVA was used to compare body water deuterium enrichments [OMNI1 compared with VEG1, and time (days: 1–4) (with random effects included to account for repeated measures)] during the nutritional intervention. A repeated measures 3-factor ANOVA (OMNI1 compared with VEG1, pre compared with post and rested compared with exercised leg) was used to compare myofibrillar protein-bound [^2^H] alanine enrichments. Repeated measures 2-factor (OMNI1 compared with VEG1 and rested compared with exercised leg) ANOVA, Bonferroni adjusted *post hoc* independent samples *t*-tests (to detect differences in the rested and exercised leg between groups), and paired *t*-tests (to detect differences in the rested and exercised leg within groups) were used to compare MyoPS rates.

For Phase 2, independent samples *t*-tests were used to compare the baseline measures of lean mass, muscle volume, muscle fiber CSA, and muscle strength. Two-factor repeated measures ANOVAs (OMNI2 compared with VEG2, and time) were used to compare temporal changes in lean mass, muscle volume, and muscle strength. Mixed-effects model ANOVAs [OMNI2 compared with VEG2, and time (with random effects included to account for repeated measures)] were used to compare diets and changes in muscle fiber CSA during the intervention period. Independent samples *t*-tests were used to compare percentage changes in muscle strength pre to post-training. When a significant group by the time interaction was found, Sidak or Tukey post hoc tests were applied to locate individual differences. Statistical significance was set at *P <* 0.05.

In Phase 1, MyoPS rates represent the primary outcome, with all other measures representing secondary outcomes. In Phase 2, lean mass represents the primary outcome, with all other measures representing secondary outcomes.

## Results

Participants’ characteristics for both phases of the study are provided in Table 1, with recruitment and allocation workflow presented in Figure 1. Twenty subjects continued from Phase 1 to Phase 2 of the study, 1 participant was excluded from Phase 2 analysis due to ill health (unrelated to the study), and 1 participant withdrew from Phase 2 because of personal reasons. In total, 21 participants completed Phase 1 of the study, with metabolic data available for 16 participants, and 22 completed Phase 2 of the study. Baseline age, body mass, and BMI did not differ at baseline in either Phase 1 or Phase 2 of the study (all *P >* 0.05), and the groups were well balanced for sex. No differences in total work performed during the experimental resistance exercise bouts in Phase 1 (37,911 ± 10,661 J in OMNI1 compared with 37,897 ± 8624 J in VEG1; *P =* 0.99), or in fatigue during each trial (all *P >* 0.05) or over the wk (*P =* 0.63) were detected between groups.

### Habitual diet

For both Phases 1 and 2, the habitual diet did not differ between groups for energy, protein, fat or carbohydrate intakes (all *P >* 0.05) (TABLE 2, TABLE 3). In Phase 1, carbohydrate, fat, and fiber intakes increased from participants’ habitual diets to the diet they received during the intervention (all *P <* 0.05). In phase 2, intakes increased with the intervention across all variables (all *P <* 0.05).

**TABLE 3:** The nutritional content of the participants’ diets during Phase 2 of the study

A	OMNI2	VEG2	*P*	
	(*n* = 12) 6 f 6 m	(*n* = 10) 5 f 5 m	Group effect	Intervention effect
Habitual diet				
Energy (MJ·d^−1^ (kcal·d^−1^)	10.3 ± 2.5 (2464 ± 594)	9.3 ± 2.7 (2217 ± 640)	0.396	—
Protein (g·d^−1^)	111 ± 40	110 ± 53	0.940	—
Protein (g·kg bm^−1^·d^−1^)	1.5 ± 0.4	1.6 ± 0.6	0.780	—
CHOs (g·d^−1^)	275 ± 58	270 ± 84	0.878	—
Fat (g·d^−1^)	94 ± 37	74 ± 22	0.166	—
Fiber (g·d^−1^)	27 ± 13	38 ± 14	0.083	—
Intervention diet				
Energy (MJ·d^−1^ (kcal·d^−1^)	12.1 ± 2.0 (2882 ± 486)	11.9 ± 2.7 (2838 ± 642)	0.861	<0.0001
Protein (g·d^−1^)	163 ± 34	146 ± 31	0.221	<0.0001
Protein (g·kg bm^−1^·d^−1^)	2.3 ± 0.3	2.1 ± 0.2	0.090	<0.0001
CHOs (g·d^−1^)	304 ± 56	314 ± 83	0.732	0.017
Fat (g·d^−1^)	101 ± 23	102 ± 25	0.861	0.005
Fiber (g·d^−1^)	27 ± 9	75 ± 12	<0.0001	<0.0001

Values represent mean ± SD. Participants underwent 10 wk of high-volume resistance exercise training with dietary intervention, alongside DXA and MRI scans, muscle biopsies, and strength testing, at regular intervals to characterize resistance exercise-induced muscle adaptations. In OMNI2, participants consumed an omnivorous diet with most of the protein coming from animal-derived sources. In VEG2, participants consumed a diet with the majority of dietary protein coming from non-animal sources. Participants in OMNI2 received a milk protein supplement (47-g protein daily), and participants in VEG2 received a mycoprotein supplement (46-g protein daily). Dietary data were analyzed with repeated measures 2-factor ANOVA. f, female; m, male; OMNI, omnivorous; VEG, vegan.

### Phase 1 dietary intervention

Dietary intake during the intervention period is displayed in Table 2. Body mass did not change over the intervention period in either group (*P >* 0.05) indicating energy balance was likely achieved. During the intervention period, energy (*P =* 0.82), fat (*P =* 0.88), and carbohydrate (*P =* 0.81) intakes did not differ between groups, and by design, daily protein intake was identical between groups (*P* = 0.67). Fiber intake was approximately double in VEG1 compared with OMNI1 (*P<*0.0001) group, as a result of the high fiber content of mycoprotein.

In OMNI1, of the 131 ± 20-g protein consumed per day, 89 ± 14-g was provided by animal-derived sources and 42 ± 8-g from non-animal sources, corresponding to 68 ± 2% and 32 ± 2% from animal and non–animal-derived sources, respectively. Meat products provided 40 ± 1-g and dairy products (including the milk protein supplement) provided 49 ± 13-g protein per day, corresponding to 32 ± 5% and 37 ± 5% of total protein, respectively. The milk protein supplement alone provided 31 ± 0-g protein per day (24 ± 4% of total protein). In VEG1, of the 127 ± 16-g protein consumed per day, 68 ± 7-g protein was derived from mycoprotein (28 ± 7-g mycoprotein-containing products, and 40 ± 3-g from supplementary isolated mycoprotein) corresponding to 54 ± 4% total protein intake. Remaining protein was provided by wheat and potato protein also contained in mycoprotein containing vegan products (17 ± 4-g protein per day, and 20 ± 2% of total protein), and from the protein present in the other elements of the diet. Overall, mycoprotein-containing products provided 46 ± 11-g daily protein and accounted for 36 ± 5% total daily protein intake.

### Body water deuterium enrichments

Saliva deuterium enrichments throughout the experimental protocol are shown in Supplemental Table 2. Body water deuterium enrichments increased from baseline (time effect; *P <* 0.0001), with no differences between groups (treatment and treatment × time interaction both *P >* 0.05).

### Daily MyoPS rates

**FIGURE 3:**
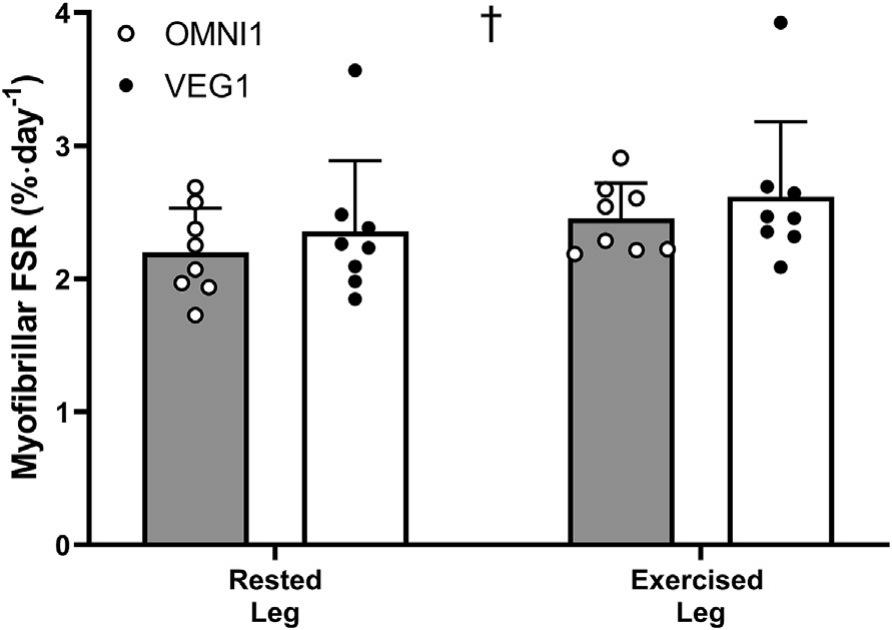
Daily free-living myofibrillar protein synthesis rates (MyoPS) during Phase 1 of the study, calculated from the body water deuterium precursor pool in 16 healthy adults consuming a 3-d fully controlled eucaloric high-protein (1.8 g·kg bm^−1^·d^−1^) diet, where the protein was provided predominantly from animal (OMNI1; *n* = 8) or exclusively non-animal (VEG1; *n* = 8) sources, in a rested and exercised (single bout of 5 × 30 maximal unilateral isokinetic knee extension contractions on 3 consecutive days) muscle. Values are means, with a standard deviation represented by vertical bars, and individual data points embedded alongside. Data were analyzed with a 2-factor ANOVA, independent samples t-tests, and paired t-tests † indicate the main effect of exercise (*P* < 0.05). The exercise effect; *P* < 0.001, treatment effect; *P* = 0.4647, treatment × exercise interaction effect; *P* = 0.9621. OMNI, omnivorous; VEG, vegan.

Myofibrillar protein-bound [^2^H] alanine enrichments are shown in Supplemental Table 2. Myofibrillar protein-bound [^2^H] alanine enrichments increased over time (*P <* 0.0001; time × leg interaction; *P =* 0.0079), in rested (OMNI1 and VEG1 both *P <* 0.0001), and in exercised (OMNI1 and VEG1 both *P <* 0.0001) muscle, with no differences between the groups (all OMNI1 compared with VEG1 interactions; *P >* 0.05). Daily MyoPS rates (Figure 3) were 13 ± 18% and 12 ± 9% greater in the exercised compared with rested leg (2.20 ± 0.33%·d^−1^ compared with 2.46 ± 0.27%·d^−1^ and 2.36 ± 0.53%·d^−1^ compared with 2.62 ± 0.56%·d^−1^) in OMNI1 and VEG1 groups, respectively (*P <* 0.001). Daily MyoPS rates did not differ between groups in either rested (*P =* 0.49) or exercised (*P =* 0.47) muscle.

### Phase 2 dietary intervention

Dietary intake during the intervention period is displayed in Table 3. The diet was well adhered to, with participants providing the required dietary records in 99.1% of cases. Protein targets were met throughout the intervention, with 83% of participants consuming ≥2 g·kg bm^−1^·d^−1^ and 100% consuming >1.8 g·kg bm^−1^·d^−1^ in OMNI2 group, and 70% of participants consuming ≥2 g·kg bm^−1^·d^−1^ and 100% consuming >1.8 g·kg bm^−1^·d^−1^ in VEG2 group. By design, protein, fat, and carbohydrate (and thus total energy) intakes all increased between participants’ habitual and intervention diets (all *P* < 0.05). Daily energy intake did not differ between groups when expressed in absolute (*P =* 0.89) or relative (*P =* 0.97) terms, the same being true for daily fat (*P =* 0.87) and carbohydrate intake (*P =* 0.70). Neither absolute daily protein intake (*P =* 0.22) nor daily protein intake relative to body mass (*P =* 0.10) differed between the groups. Daily energy, protein, fat, and carbohydrate intakes all remained consistent throughout the intervention period, when expressed in both absolute terms and relative to body mass, after the same temporal pattern in both groups (time, and treatment × time effects; all *P >* 0.05). A subset analysis (*n* = 3 in each group) was carried out to estimate from which sources daily protein was obtained. In OMNI2, 67.1% was obtained from animal sources and 32.9% was obtained non-animal sources. In VEG2, 99.4% was obtained from non-animal sources and 0.6% coming from other animal sources.

### Body composition

**FIGURE 4:**
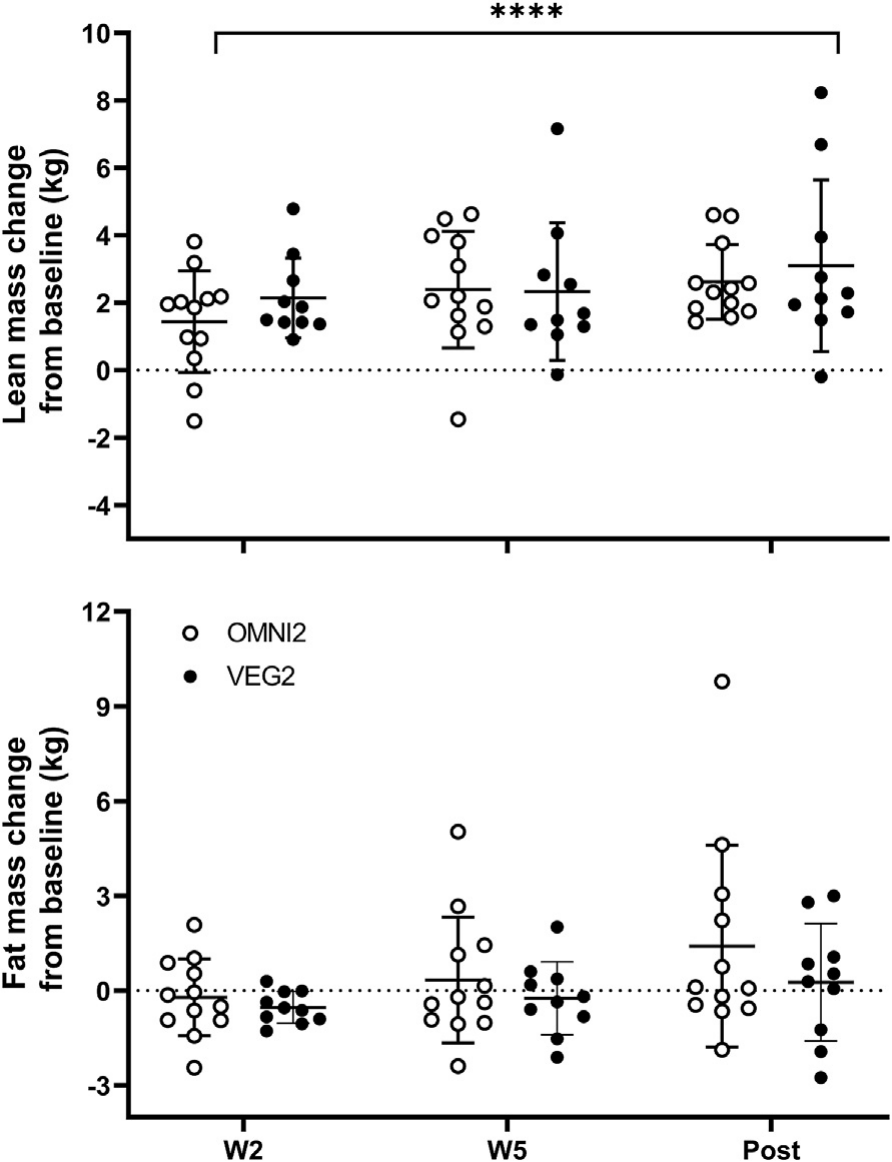
Whole-body lean mass and fat mass change (kg) in response to 10 wk of resistance exercise training in healthy young men and women consuming either a high-protein omnivorous diet (OMNI2; *n* = 12) or a majority non–animal-derived diet (VEG2; *n* = 10). Values are mean ± SDs, with individual data points embedded alongside. Data were analyzed with 2-factor ANOVAs. ∗Indicates the main effect of time. No differences were observed between the groups. Whole-body lean mass; time effect; *P* < 0.0001, treatment effect; *P* = 0.9428, treatment × time interaction effect; *P* = 0.6124. fat mass; time effect; *P* = 0.0612, treatment effect; *P* = 0.3555, treatment × time interaction effect; *P* = 0.4946. OMNI, omnivorous; VEG, vegan.

Whole-body lean mass and fat mass throughout the experiment are displayed in Figure 4. At baseline, neither lean nor fat mass was different between groups (*P >* 0.05). Fat mass remained unchanged throughout the experiment (time, treatment, and treatment × time effects; *P >* 0.05). Lean mass increased with training (from 50.5 ± 3.6 and 50.6 ± 3.7 kg) in a relatively linear fashion to 53.1 ± 3.7 kg and 53.7 ± 4.4 kg in OMNI2 and VEG2, respectively, post-training (time effect; *P <* 0.0001), with no differences between groups (treatment and treatment × time effects; *P >* 0.05). Total lean mass increased by 5.4 ± 0.8% in OMNI2, and 5.6 ± 1.2% in VEG2, with this delta change also not different between the groups (*P =* 0.9298). The increase in lean mass was apparent in the legs (OMNI2, 4.7 ± 1.4%; VEG2, 5.0 ± 1.3%), arms (OMNI2, 10.6 ± 1.4%; VEG2, 10.2 ± 1.8%), and trunk (OMNI2, 5.2 ± 1.3%; VEG2, 5.3 ± 1.4%), with the percentage change greater in the arms than legs (*P <* 0.001) and trunk (*P <* 0.01) and no differences between groups (treatment and treatment × region effects; *P >* 0.05).

### Skeletal muscle size

**FIGURE 5:**
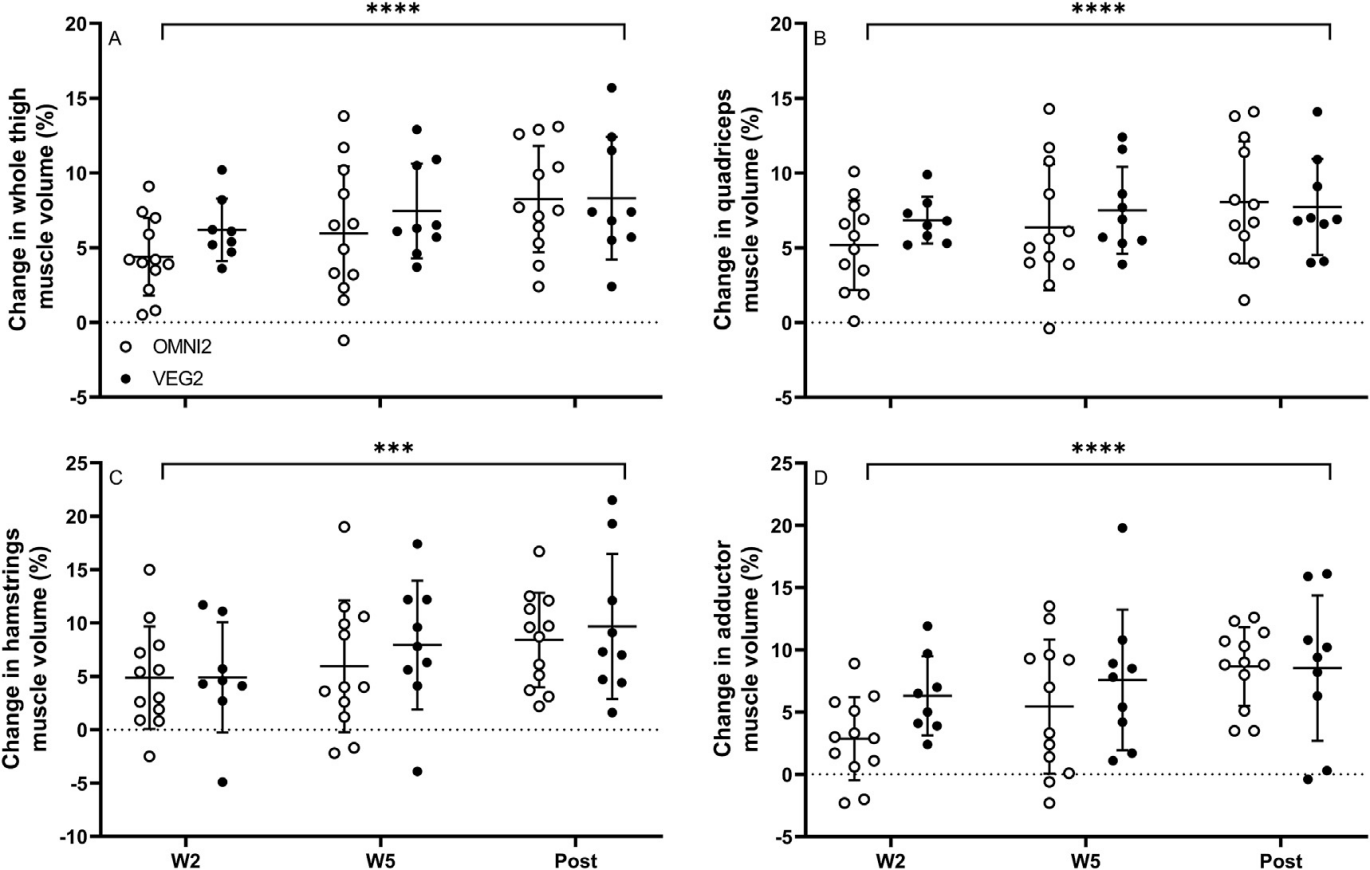
The whole thigh (A), quadriceps (B), hamstring (C), and adductor (D) muscle volumes at baseline (pre) and following 2, 5, and 10 wk of resistance exercise training (5 times per wk) in healthy young men and women consuming either a high-protein omnivorous diet (OMNI2; *n* = 12) or a majority non–animal-derived diet (VEG2; *n* = 9). Values are mean ± SDs, with individual data points embedded alongside. Data were analyzed with 2-factor ANOVAs. ∗ Indicates the main effect of time. No differences were observed between the groups for any of the variables. The time effect; A; *P* < 0.0001, B; *P* < 0.0001, C; *P* < 0.001, D; *P* < 0.0001. The treatment effect : A; *P* = 0.9688, B; *P* = 0.9994, C; *P* = 0.7671, D; *P* = 0.9434. Treatment × Time interaction effect; A; *P* = 0.7099, B; *P* = 0.7103, C; *P* = 0.7854, D; *P* = 0.5752. OMNI, omnivorous; VEG, vegan.

The whole thigh, quadriceps, hamstring, and adductor muscle volumes determined through MRI throughout the experiment are displayed in Figure 5. At baseline, no significant differences between the groups were observed in any of these parameters (all *P >* 0.05). The whole thigh (OMNI2 Δ, 253 ± 147 cm^3^; VEG2 Δ, 269 ± 193 cm^3^), quadriceps (OMNI2 Δ, 125 ± 79 cm^3^; VEG2 Δ, 127 ± 83 cm^3^), hamstring (OMNI2 Δ, 55 ± 36 cm^3^; VEG2 Δ, 64 ± 53 cm^3^), and adductor (OMNI2 Δ, 73 ± 40 cm^3^, VEG2 Δ, 77 ± 65 cm^3^) muscle volumes increased with training, from pre to post, in both groups (time effect; *P <* 0.001) with no differences between groups (treatment and treatment × time effects; *P >* 0.05). Likewise, no significant differences in the whole thigh (OMNI2, 8.3 ± 3.6%; VEG2, 8.3 ± 4.1%), quadriceps (OMNI2, 8.1 ± 4%; VEG2, 7.7 ± 3.2%), hamstring (OMNI2, 8.4 ± 4.4%; VEG2, 9.7 ± 6.8%) or adductor (OMNI2, 8.7 ± 3.2%; VEG2, 8.5 ± 5.8%) muscle-volume percentage change was observed between groups (all *P >* 0.05). Each muscle group was hypertrophied to a similar extent (*P >* 0.05).

### Skeletal muscle fiber size

**FIGURE 6:**
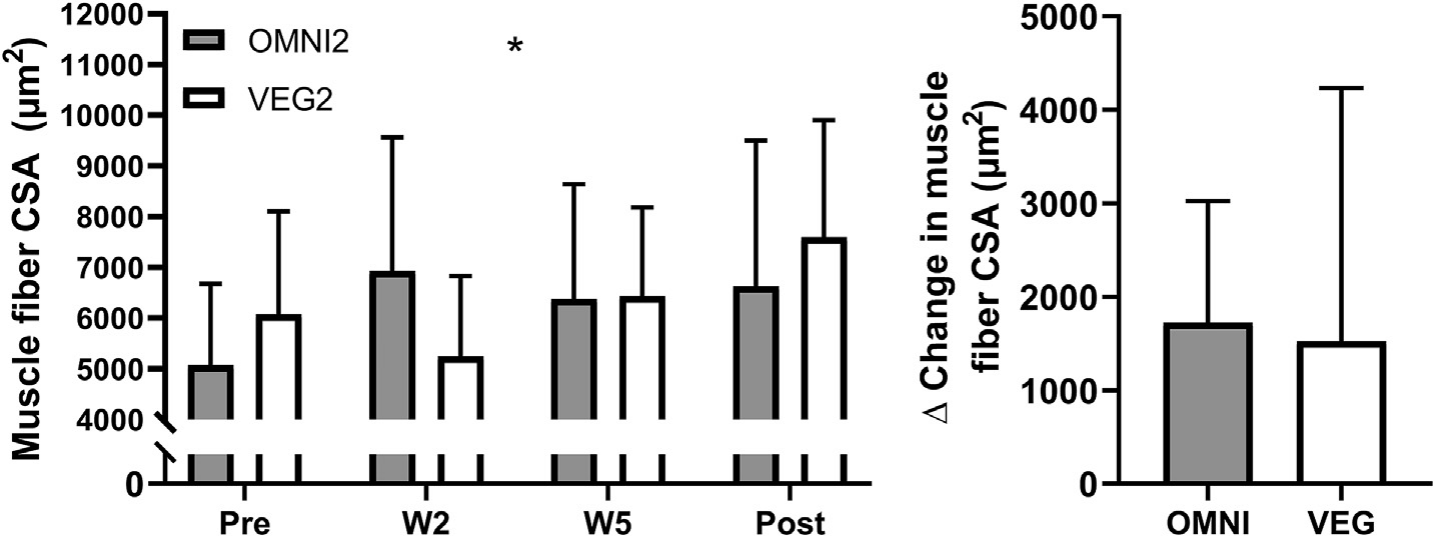
Mean muscle fiber cross-sectional area and the change in muscle fiber cross-sectional area in response to 10 wk of resistance exercise training in healthy young men and women consuming either a high-protein omnivorous diet (OMNI2; *n* = 8) or a majority non–animal-derived diet (VEG2; *n* = 8). Values are means ± SDs. Data were analyzed with a mixed-effects model ANOVA. ∗ Indicates the main effect of time. No differences were observed between the groups. The time effect; *P* < 0.05, treatment effect; *P* = 0.9760, treatment × time interaction effect; *P* < 0.05. CSA, cross-sectional area; OMNI, omnivorous; VEG, vegan.

Data are displayed for an *n* of 16 [OMNI2 (*n =* 8), VEG2 (*n =* 8)], and the mean number of fibers counted in the analyses were 186 ± 116. At baseline, muscle fiber types were distributed as follows: 51 ± 8% for type I and 49 ± 8% for type II in OMNI2, and 47 ± 15% for type I and 53 ± 15 for type II in VEG2. Fiber type distribution did not change with training (time effect *P* > 0.05) and did not differ between the groups (*P* > 0.05). Mean muscle fiber CSA throughout the experiment are displayed in Figure 6. At baseline, no significant differences in mean, type I, or type II muscle fiber CSAs were observed between the groups (*P >* 0.05). Mean (OMNI2 Δ, 1724 ± 1299 μm^2^; VEG2 Δ, 1522 ± 2709 μm^2^) and type II (OMNI2 Δ, 1907 ± 1362; VEG2 Δ, 1476 ± 2693 μm^2^) muscle fiber CSA increased with training, from pre to post, in both groups (time effects; *P =* 0.0147 and *P =* 0.0116, respectively). There were treatment × time effects for both mean (*P =* 0.0211) and type II muscle fiber CSA (*P =* 0.0334), both increasing with training but in different temporal patterns. No significant differences in mean (OMNI2, 33 ± 24%; VEG2, 32 ± 47%), type I (OMNI2, 28 ± 24%; VEG2, 24 ± 50%), or type II (OMNI2, 33 ± 20%; VEG2, 32 ± 50%) muscle fiber CSA percentage increases, from pre to post, were observed between groups over the 10 wk of training (*P >* 0.05).

### Muscle strength

**FIGURE 7:**
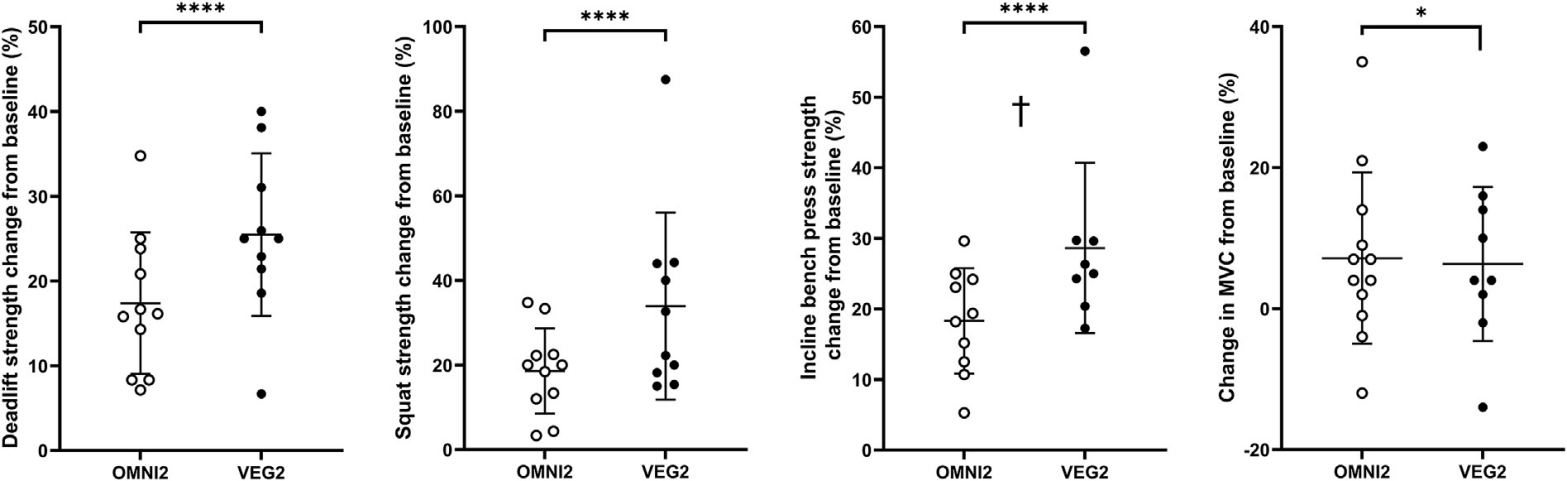
The percentage change in 1-repetition maximum strength in the deadlift, barbell back squat, incline bench press, and knee extensor peak isometric torque (MVC) after 10 wk of resistance exercise training in healthy young men and women consuming either a high-protein omnivorous diet (OMNI; *n* = 11) or a majority non–animal-derived diet (VEG; *n* = 9). Values are mean ± SDs, with individual data points embedded alongside. Data were analyzed with 2-factor ANOVAs. The main effect of training was present for all 3 exercises (*P* < 0.05). ∗ Indicates the main effect of time. † Indicates a significant difference between groups (*P* < 0.05). The time effect; deadlift; *P* < 0.0001, squat; *P* < 0.0001, incline bench press; *P* < 0.0001, MVC; *P* < 0.05. Treatment effect; deadlift; *P* = 0.7087, squat; *P* = 0.7271, incline bench press; *P* = 0.7429, MVC; *P* = 0.6981. treatment × time; deadlift; *P* = 0.1396, squat; *P* = 0.2234, incline bench press; *P* = 0.1862, MVC; *P* = 0.9693. MVC, maximal voluntary contraction; OMNI, omnivorous; VEG, vegan.

Strength data before and after training are displayed in Figure 7. At baseline, there were no differences between the groups in strength for the deadlift (*P =* 0.4945), squat (*P =* 0.5438), or incline bench press (*P =* 0.6091) exercises. Strength increased after training in all 3 of these measures (time effects; all *P <* 0.0001), with no differences between groups (all *P >* 0.05) or interaction effects (all *P >* 0.05). The percentage increase in strength also did not differ between groups for the squat (*P =* 0.1775) but trended to be higher in VEG2 compared with OMNI2 for the deadlift (*P =* 0.0526) and reached a significantly greater response in VEG2 than OMNI2 for the incline bench press (*P <* 0.05). Knee extensor peak isometric torque increased with training, from 225 ± 17 N⋅m to 239 ± 18 N⋅m in OMNI2, and 215 ± 22 N⋅m to 228 ± 24 N⋅m in VEG2 (time effect; all *P <* 0.0001), with no differences between groups (all *P >* 0.05) or interaction effects (all *P >* 0.05).

## Discussion

In the current study, we demonstrate that a high-protein (1.8 g·kg bm^−1^·d^−1^), mycoprotein-rich, non–animal-derived diet can facilitate comparable daily, free-living MyoPS rates compared with an isonitrogenous omnivorous diet in both rested and exercised muscle in healthy young adults. Moreover, this translated to the (high) protein-matched omnivorous and (virtually exclusively) non-animal diets supporting comparable increases in skeletal muscle fiber size, muscle volume, lean mass, and strength throughout 10 wk of high-volume (5 times per wk) resistance training.

Dietary protein intake and resistance exercise are the key drivers of skeletal muscle hypertrophy, achieved physiologically by additively stimulating MyoPS rates and thereby facilitating periods of positive net protein balance [1,2,50]. The summative impact of nutrition and resistance exercise has been shown in the hours after a bout of exercise [50], and recent data show that the magnitude of post-exercise *daily* MyoPS rates are predictive of the level of hypertrophy if training persists (once initial perturbations to novel training stimuli have been accounted for) [8,51,52]. Here we showed that high protein diets sustained relatively high daily, free-living MyoPS rates at the outset of training that were potentiated by approximately 11% with daily resistance exercise (Figure 3). These data are indicative of the mechanistic underpinning for why protein intakes >1.6 g/kg bm (well above the RDA of 0.75–0.8 g/kg bm in the United States/United Kingdom) [9] in concert with progressive, high-volume resistance training, is the optimal strategy to support maximal rates of muscle hypertrophy [10,53,54]. Indeed, in the present work, we report that applying this strategy for 10 wk translated to a considerable degree of muscle hypertrophy (FIGURE 4, FIGURE 5, and 6); specifically, a ∼2.8 kg (5.5%) increase in whole body lean mass, and ∼8.3% and ∼32% growth of thigh muscle volume and mean muscle fiber CSA, respectively, across groups. Our temporal assessments allowed us to discern that this muscle hypertrophy was relatively linear throughout training, occurring at rates of 0.55%, 0.83% and 3.2% per wk for lean mass, thigh volume, and muscle fiber CSA, respectively. When delineated by biological sex (for the purpose of allowing comparison of our mixed-sex population to analogous studies involving single-sex cohorts), we observed increases in lean mass of 3.6 kg (0.36 kg per wk) and 2.1 kg (0.21 kg per wk) in men and women, respectively. The temporal and total hypertrophy we observed is toward the upper end of what may be expected given the previous analogous studies [that is, studies adopting diets containing 1.5–1.9 g·kg bm^−1^·d^−1^ protein, and resistance training for 12 wk (3–5 sessions per wk)] which have observed increases in lean mass of 1.9 kg (0.16 kg·wk) and 3.9 kg (0.33 kg·wk) in young men [16,55] and 1.9 kg (0.16 kg·wk) in young women [49]. It is evident, therefore, that high rates of daily MyoPS were sustained through the intervention to facilitate a persistent positive net muscle protein balance and drive high rates of hypertrophy, as indicated by multiple measurements varying in scope from the whole-body to myocellular level.

Literature surrounding protein requirements to support resistance training-induced muscle hypertrophy has largely been deduced from studies where participants have consumed omnivorous diets with an emphasis on the intake of (generally supplemental) high-quality animal-derived proteins [10]. It has been proposed that non–animal-derived dietary proteins may be inferior in their capacity to support muscle protein synthesis rates, and, therefore, training-induced increases in muscle mass [[15], [16], [17],^56^,^57^]. This would imply that current protein recommendations may not translate to those adhering to a non–animal-derived diet. In the present study, and in line with our previous work in older adults [23], we show that participants adhering to either an omnivorous or vegan high protein (1.8 g·kg bm^−1^·d^−1^) diet displayed comparable daily free-living MyoPS rates in rested and exercised muscle (Figure 3). Importantly, these analogous MyoPS rates preceded, and translated to, comparable increases in muscle mass over a prolonged free-living resistance training period, irrespective of whether protein was subsequently obtained from omnivorous (that is, 67% animal, 29% non-animal) or non-animal (that is, <1% animal, 99% non-animal) sources. Indeed, the hypertrophic response was comparable across all indices of muscle size [5.4% (2.6 kg) compared with 5.6% (3.1 kg), 8.3% compared with 8.3%, and 43% compared with 32% increases in response to the omnivorous and vegan diets for lean mass, thigh volume, and muscle fiber CSA, respectively] and also remarkably similar temporally across groups (FIGURE 4, FIGURE 5, FIGURE 6).

The apparent contrast between our data and previous narratives of non–animal-derived dietary proteins being inferior to support MyoPS rates and associated adaptive responses may be explained in several ways. Previous studies have generally investigated a minimal number of (lower quality) supplemental (that is, refined/isolated) plant-based proteins for their capacity to stimulate hourly (post-exercise) MyoPS rates [15,56]. Fewer data still are available concerning vegan *diets* and *daily* MyoPS rates. Here we demonstrated that the use of mycoprotein-containing products and supplements can feasibly be used as a high-quality foundation for the construction of a non–animal-derived diet to facilitate high rates of MyoPS and, therefore, hypertrophy in resistance training young adults. This is in line with our previous work in which we demonstrated that mycoprotein ingestion robustly stimulates hourly post-exercise MPS rates [22], and can be incorporated into a high-protein non–animal-derived diet (as food products and supplemental drinks) to support high daily MyoPS rates in older adults [23]. It is likely that the close link between daily MyoPS rates and adaptive responses observed in this study is explained by the former being determined under free living conditions over several days, and thus encapsulating multiple feeding-fasting cycles to the diet in its entirety, while also incorporating habitual activity and diurnal variation. Indeed, a collection of recent studies have all shown that daily measurements of post-exercise free-living MyoPS rates can accurately predict the magnitude of hypertrophy on an individual level if training persists [8,51]. Moreover, having multiple meals containing differing protein sources (VEG2 contained potato, wheat, and other protein sources alongside the predominant mycoprotein component) may also aid in providing a sufficiently diverse array of amino acids to avoid limitations to muscle protein anabolism relating to bioavailability or individual amino acid deficiencies. Whether our findings would hold true if we had predicated the vegan diet on other dietary protein options is unclear and remains an area ripe for future investigation.

Another key consideration for why our 2 dietary groups achieved comparable responses to training concerns the high(er) protein diets selected. We deliberately selected an “optimal” training and dietary approach to maximize MyoPS rates and training adaptations within our control group, and therefore to reveal any potential decrements in the vegan diet. As a result, we cannot extrapolate the present work to low(er) protein diets more in line with current RDAs; although based on the hourly post-exercise MPS response to bolus mycoprotein ingestion [22], it seems reasonable to expect a similar result. In line with our findings, it has been shown that participants adhering to resistance training increased muscle mass comparably when supplemented with pea or whey protein [20], with rice or whey protein [58], when consuming a lacto-ovo-vegetarian or beef-containing diet [59], or when consuming a habitual omnivorous and vegan diet [21]. Our data therefore support and extend upon an emerging evidence base that suggests non–animal-derived diets may be equivalently capable of supporting skeletal muscle adaptive responses to resistance training assuming sufficient protein is consumed. Significantly, we arrive at this conclusion in the context of largely self-selected (although directed and counseled) free-living *diets* (as opposed to the manipulation of supplemental protein alone). An important pragmatic and translational point here is that many individuals pursuing resistance-training induced increases in muscle mass will likely follow a high(er) protein diet in line with the present study and those described above; in fact, our subjects habitually consumed significantly above the RDA (∼100% above), and only 40% less than that during the study (Table 3).

During Phase 2 of the study, significant muscle hypertrophy occurred alongside considerable increases in strength (23% on average across exercises), the magnitude of which is in line with previous comparable studies [49,55]. These measures were more variable than those of muscle mass (ranging from 4% to 88% for strength compared with 0 to 14% and 2% to 16% for lean mass and whole thigh volume, respectively), which likely related to the multifaceted nature of adaptive changes in muscle strength, especially so with compound, multi-joint exercises. Although being related [60], increases in muscle strength are not just contingent upon increased muscle size, but also a complex array of neuromuscular adaptations, alongside improvements in technical proficiency and skill acquisition [61,62], even in trained individuals [[63], [64], [65], [66]]. The present work shows that any such contributions to adaptive increase in muscle strength are not adversely affected by a well-designed vegan diet. In fact, a single index of strength (incline bench press) actually increased to a greater degree in the vegan group, possibly an artifact of a single “high responder” (56% increase; also disproportionately improving strength in the squat by 88%). We therefore concluded that the experimental approach adopted, albeit within the constraints of its design, comprehensively translated our past [22,23] and present findings to show that the dietary protein source did not appreciably affect the “mechanisms to movement” of human muscle adaptation to resistance exercise.

There are some ancillary considerations of our data that we would be remiss not to discuss. The current study applied a holistic dietary intervention as opposed to protein supplementation (alone), meaning aspects beyond protein *per se* require consideration. Aside from being protein rich, meat and dairy are nutrient-dense whole food groups capable of fulfilling several micronutrient needs relevant to both health and training adaptations. To assess the role of dietary protein origin *per se,* we chose to supplement all participants with creatine to avoid the most obvious and consequential (to training adaptations) [[67], [68], [69]] deficiency a vegan diet may create [40,41]. We also reasoned that this could be considered part of an optimal approach [[67], [68], [69]], accessible to omnivores and vegans alike, although it is likely to have amplified increases in lean mass, particularly in the early phase of the study, which is a consideration when interpreting our results. However, there is an array of other micronutrient intakes that likely differ between omnivorous and vegan diets (for example, vitamin B12, iron, B-alanine, omega fatty acids, carnitine) [70] that we did not control for, or measure, that may modulate (more prolonged and sustained) optimal muscle adaptive responses and/or overall health. A practical consideration was the feasibility of a high(er) protein diet within the vegan group. Although we (more than) achieved our target protein intake in both groups, demonstrating feasibility, achieving protein targets was anecdotally more difficult for participants in the vegan group (expressed during regular dietary counseling visits). This appears to be supported by the literature, wherein those adhering to vegan and vegetarian diets habitually consume lower protein [[71], [72], [73]] and may ultimately represent a practical barrier to be overcome within vegan sports nutrition. A further important consideration for the present work is that although we showed no clear physiological differences between the two intervention diets, in either the acute or chronic stage, the study was not designed to test statistical equivalency. As such, future trials might use equivalency testing, with a large sample size, to more properly examine subtle differences between dietary patterns, and the extent of their parity. Finally, from a statistical perspective, we acknowledge the increased risk of making a type 1 error due to multiple testing outcomes required to comprehensively measure muscle growth.

To conclude, we showed that a high protein, mycoprotein-rich vegan diet can support comparable daily free-living MyoPS rates as an isonitrogenous omnivorous diet in resistance training young men and women. These metabolic data translated to (and likely explain) the omnivorous and (virtually exclusive) vegan diets facilitating comparable increases in muscle fiber size, muscle volume, whole body lean mass, and muscle strength throughout 10 wk of high-volume resistance training. As such, a carefully designed vegan diet is capable of supporting optimal skeletal muscle adaptive responses to resistance training.

## Data Availability

Data described in the manuscript may be made available upon request, pending application.

## Appendix A Supplementary data

Multimedia component1 Multimedia component1
