# Supplementary material for: Vegan and Omnivorous High Protein Diets Support Comparable Daily Myofibrillar Protein Synthesis Rates and Skeletal Muscle Hypertrophy in Young Adults
Source: J Nutr. 2023 Feb 22;153(6):1680–95. doi: 10.1016/j.tjnut.2023.02.023 (PMC10308267; doi:10.1016/j.tjnut.2023.02.023)
Supplement: Multimedia component1 [file mmc1.pdf]

VEGAN AND OMNIVOROUS HIGH PROTEIN DIETS SUPPORT EQUIVALENT DAILY MYOFIBRILLAR PROTEIN SYNTHESIS RATES AND SKELETAL MUSCLE HYPERTROPHY IN YOUNG ADULTS: A RANDOMIZED CONTROLLED TRIAL.

Alistair J Monteyne[1], Mariana OC Coelho[1], Andrew J Murton[2,3], Doaa R Abdelrahman[2,3], Jamie R Blackwell[1], Christopher P Koscien[1], Karen M Knapp[4], Jonathan Fulford[4], Tim JA Finnigan[5], Marlou L Dirks[1], Francis B Stephens[1], Benjamin T Wall[1]

SUPPLEMENTARY MATERIAL

Blood Sampling

Participants reported to the laboratory on a designated day each week for the collection of a fasting blood sample. Blood samples were obtained via venepuncture, with 8 mL of venous blood collected into liquid heparin-containing tubes (BD vacutainer LH; Becton, Dickinson and Company) and centrifuged immediately (3000 × g, 4◦C, 10 min). Blood plasma was aliquoted and frozen at –80◦C for subsequent analysis. A further 6 mL of blood was collected into SST vacutainers (BD vacutainers SST II, Becton, Dickinson and Company) which were left to clot at room temperature for ≥30 min and then centrifuged (3000 × g, 4◦C, 10 min) to obtain blood serum. Serum was aliquoted before freezing at –80◦C for subsequent analyses.

Body water deuterium enrichment

Body water deuterium enrichment was measured using the saliva samples collected daily throughout the study, at the University of Texas Medical Branch. A ThermoFisher Delta V Advantage Isotope Ratio mass spectrometer (IRMS) (Bremen, Germany), equipped with a Finnigan GasBench II (Thermo Fisher Scientific, Waltham, MA, USA), was used for hydrogen/deuterium ratio measurements. After uncapping a 12 mL Exetainer (Labco Limited,

Lampeter, UK), 5 mg of activated charcoal (Thermo Fisher Scientific) and 200 mg of copper powder (Thermo Fisher Scientific) were introduced into the Exetainer followed with a platinum catalytic rod (Thermo Fisher Scientific). The activated charcoal and copper powder were added to remove any potential contaminants in the samples that might poison the platinum catalyst. After putting 200 uL of sample into the Exetainer, the Exetainer was recapped and placed into the GasBench II and flushed with 2% H_2 in helium for 7 min. The sample was allowed to equilibrate with the flushed gas at room temperature (~4-6 h). At the end of the equilibration, an aliquot of the headspace in the Exetainer was injected into the Thermo Delta V Advantage mass spectrometer system for stable hydrogen isotope ratio measurement against the reference gas H_2. A standard calibration curve was prepared using 99.9% deuterium-enriched water (Sigma-Aldrich, St. Louis, MO, USA), and the deuterium (^2H) enrichment in duplicate saliva samples was determined (1).

Myofibrillar bound ^2H alanine enrichments

Myofibrillar protein-enriched fraction was extracted from ~50 mg of wet weight muscle tissue by hand-homogenisation on ice using a pestle in a standard homogenisation buffer (TRIS-HCl 50 mM, EDTA 1 mM, EGTA 1 mM, β-glycerophosphate 10 mM, NaF 50 mM, activated sodium orthovanadate 0.5mM, and complete protease inhibitor cocktail tablet (Roche Holding AG, Basel, Switzerland)) (7.5 µL/mg). The samples were centrifuged at 2,200 g for 10 min at 4°C, the pellet was then washed with 500 µL of homogenisation buffer, and centrifuged at 700 g for 10 min at 4°C. The myofibrillar protein was solubilised by adding 750 µL of 0.3 M NaOH and heating for 30 min at 50°C with samples being vortexed every 10 min. Samples were then centrifuged for 10 min at 10,000 g and 4°C, the supernatant containing the myofibrillar protein was kept and the collagen protein pellet was discarded. The myofibrillar proteins were precipitated by the addition of 500 µL of 1 M PCA and centrifuged at 700 g and 4°C for 10

min. Myofibrillar proteins were then washed with 70% ethanol twice and hydrolysed overnight in 2 mL of 6 M HCl at 110°C. The free amino acids from the hydrolysed myofibrillar protein pellet were dried under vacuum with a Speed-Vac rotary dryer (Savant Instruments, Farmingdale, NY, USA) for 3 h at 80°C radiant cover. The free amino acids were subsequently solubilized in 1.5 mL 25% acetic acid solution and passed over cation exchange AG 50W-X8 resin columns (mesh size: 100-200, ionic form: hydrogen; Bio-Rad Laboratories, Hercules, CA) and eluted with 6 M NH_4OH. Following this, the purified amino acids were dried and derivatised to tert-butyldimethylsilyl derivatives via the addition of 50 µl MTBSTFA + 1% tert-butyl-dimethylchlorosilane and 50 µl acetonitrile, which was then vortexed and heated at 95°C for 40 min. The samples were transferred to a GC vial. The level of enrichment of deuterated-alanine was analysed using a ThermoFisher Delta V Advantage Isotope Ratio mass spectrometer (IRMS) fitted with a Trace 1310 GC with an on-line high-temperature thermal conversion oven (HTC) at 1420°C. The sample (1µl) was injected in splitless mode at an injection port temperature of 250°C. The peaks were resolved on a 30m × 0.25mm ID × 0.25µm film Agilent Technologies DB-5 capillary column (temperature programme: 110°C for 1 min; 10°C·min^{-1} ramp to 180°C; 5°C·min^{-1} ramp to 220°C; 20°C·min^{-1} ramp to 300°C; hold for 2 min) prior to pyrolysis. Helium was used as the carrier gas with a constant flow of 1ml/min. Any amino acid eluting from the gas chromatograph was converted to H_2 before entry into the IRMS. The enrichment of the alanine tracer was measured by monitoring the ion masses 2 and 3 to determine the $^2H/^1H$ ratios in the samples and referenced to the calibration curve. The calibration curve consisted of a series of known concentrations of d_4-alanine and was applied to assess both the linearity of the mass spectrometer and to control for the loss of tracer. The isotopic abundances were expressed as the delta notation, δ2H per mil (‰) deviation from VSMOW (Vienna Standard Mean Ocean Water) standard (1). Values of delta per mil given by the IRMS were transformed into MPE.

Squat, deadlift and incline deadlift 1RM testing

Participants performed a warm-up with a self-selected load that allowed comfortably completion of 6-10 repetitions, a 2 min rest and then selecting a weight which allowed 3 repetitions. After a further 2 minutes rest participants were instructed to increase the load and perform a single submaximal repetition. A series of single maximum repetition attempts were then completed until a 1RM was achieved, with load increments increasing ~5-20% for each attempt, 3 minutes rest between attempts, and with 1RM achieved within 3-7 attempts. The researcher overseeing the testing ensured that proper lifting technique was demonstrated and practised throughout, noting range of motion and individual variation in technique

For the barbell back squat, participants were instructed to squat to parallel (midline of the thigh parallel with the floor). If this range of motion was not possible, they were asked to move through the maximum achievable range of motion which was then replicated throughout. Range of motion in maximum attempts was matched to that performed during the submaximal warm-up sets, such that if participants produced a range of motion significantly shorter than demonstrated previously it was deemed unacceptable. The conventional deadlift was deemed acceptable contingent on continued upward momentum until upright. The lift was deemed unacceptable if there was downward movement of the bar before completion of the lift, failure to stand erect with shoulders back, failure to lock the knees straight, and if the bar was supported on the thighs without upward momentum. For the incline bench press, participants were instructed to lower the bar as far as was comfortable, with range of motion in maximum efforts matched to submaximal efforts, and then pressing upwards until the elbows were locked out. The lift was deemed unacceptable if there was any downward movement of the bar in the course of being pressed out.

Biodex Testing

After undertaking an incremental warm-up protocol, participants completed three 3 second maximal isometric voluntary contractions (MVC) at a 70° joint angle, with peak isometric torque recorded. Participants then completed three maximal concentric isokinetic leg extension contractions at a speed of 60° per second over a central 80° range of motion using their dominant leg, with peak isokinetic torque recorded. This was followed by 5 sets of 30 repetitions of maximal concentric isokinetic leg extension contractions at a speed of 60° per second over a central 80° range of motion using their dominant leg in line with our previous work (20). Work done (J) was recorded for each completed set, and fatigue was calculated as the percentage decrement in work done between the first and last set. Verbal encouragement was provided throughout testing to encourage maximal effort on each repetition.

Table S1. Resistance training program structure.

	Order	Exercise	Sets	Notes
**Pull 1**	A.	Rope Straight Arm Pulldown	3	
	B.	Deadlift	4	
	C.	Bent Over Dumbbell Row	3	
	D.	Seated Cable Row	3	Rest-Pause
	E.	Prone Rear Delt Fly	3	
	F.	Hammer Curl	3	
	G.	Seated Dumbbell Curl	3	
	H.	Plank	3	
**Push 1**	A.	Neutral Cable Fly	3	
	B.	Low Incline Barbell Press	3	
	C.	Dumbbell Shoulder Press	3	Rest-pause
	D1.	Chest Supported Lateral Raise	3	
	D2.	Seated Lateral Raise	3	
	E.	Press Up	3	
	F.	Rope Pushdown	3	
	G.	Decline Sit Up	3	
**Lower**	A.	Unilateral Leg Curl	3	
	B.	Barbell Squat	4	
	C.	Dumbbell Romanian Deadlift	3	
	D	Leg Press	3	
	E.	Leg Extension	3	Drop set
	F.	Calf Raise	3	
	G.	Hanging Leg Raise	3	
**Pull 2**	A.	Facepull	3	
	B.	One Arm Dumbbell Row	3	Rest-pause
	C.	Pull Up	3	
	D.	Lat Pulldown	3	Drop set
	E.	Dumbbell Shrug	3	
	F1.	Dumbbell Curl	3	
	F2.	Hammer Curl	3	
	G.	Bicycle Crunch	3	
**Lower & Push**	A.	Heel Elevated Dumbbell Goblet Squat	3	Rest-pause
	B1.	Dumbbell Split Squat	3	
	B2.	Single Leg Glute Bridge	3	
	C.	Unilateral Leg Extension	3	
	D	Incline Dumbbell Press	3	
	E.	Machine Chest Press	3	
	F1.	Seated Lateral	3	
	F2.	Seated Front Raise	3	
	G.	Unilateral Pushdown	3	

Table S2. Saliva deuterium enrichments and myofibrillar [2H] alanine enrichments during Phase 1 of the study.

	OMNI1	VEG1
	**Saliva deuterium enrichments (%)**	
Day 1	0.001 (0.001 to 0.001)	0.001 (0.001 to 0.002)
Day 2	0.70 (0.56 to 0.085)	0.68 (0.55 to 0.81)
Day 3	0.69 (0.54 to 0.83)	0.69 (0.59 to 0.79)
Day 4	0.66 (0.53 to 0.79)	0.71 (0.61 to 0.80)
Day 5	0.73 (0.60 to 0.85)	0.73 (0.62 to 0.84)
	**Myofibrillar [2H] alanine enrichments [MPE]**	
Baseline	0.08 (0.07 to 0.09)	0.07 (0.05 to 0.09)
Rested	0.25 (0.21 to 0.29)	0.26 (0.21 to 0.30)
Exercised	0.27 (0.22 to 0.31)	0.28 (0.24 to 0.32)

Values represent mean and confidence intervals. OMNI, omnivorous diet; VEG, non-animal-derived diet. Participants received a 3-day controlled, eucaloric and high-protein diet, alongside daily unilateral resistance exercise, with deuterated water utilised to measure rested and post-exercise daily myofibrillar protein synthesis rates. In OMNI1, participants consumed an omnivorous diet with the majority of dietary protein coming from animal-derived sources. In VEG1, participants consumed a diet derived from non-animal sources with a large proportion of their protein coming from mycoprotein containing products and mycoprotein. Both groups received 1.8 $g \cdot kg\ bm^{-1} \cdot day^{-1}$ protein. Saliva deuterium enrichments were analyzed with a mixed-effects model ANOVA, and myofibrillar enrichements with a repeated measures three-way ANOVA.

REFERENCES

1. Wong WW, Clarke LL. A Hydrogen Gas-Water Equilibration Method Produces Accurate and Precise Stable Hydrogen Isotope Ratio Measurements in Nutrition Studies. The Journal of Nutrition 2012;142(11):2057-62. doi: 10.3945/jn.112.167957.
